# Resumption of menses in anorexia nervosa during a course of family-based treatment

**DOI:** 10.1186/2050-2974-1-12

**Published:** 2013-04-08

**Authors:** Julianne P Faust, Andrea B Goldschmidt, Kristen E Anderson, Catherine Glunz, Melanie Brown, Katharine L Loeb, Debra K Katzman, Daniel Le Grange

**Affiliations:** 1 Department of Psychiatry and Behavioral Neuroscience, The University of Chicago, 5841 S. Maryland Ave., MC3077, Chicago, IL, 60637, USA; 2 Division of Adolescent Medicine, Department of Pediatrics, The Hospital for Sick Children and University of Toronto, Toronto, Canada; 3 Department of Pediatrics, The University of Chicago, Chicago, IL, USA; 4 School of Psychology, Fairleigh Dickinson University, Teaneck, NJ, USA

**Keywords:** Return of menses, Adolescent anorexia nervosa, Family-based treatment

## Abstract

**Background:**

The resumption of menses (ROM) is considered an important clinical marker in weight restoration for patients with anorexia nervosa (AN). The purpose of this study was to examine ROM in relation to expected body weight (EBW) and psychosocial markers in adolescents with AN.

**Methods:**

We conducted a retrospective chart review at The University of Chicago Eating Disorders Program from September 2001 to September 2011 (*N* = 225 females with AN). Eighty-four adolescents (Mean age = 15.1, *SD* = 2.2) with a *DSM-IV* diagnosis of AN, presenting with secondary amenorrhea were identified. All participants had received a course of outpatient family-based treatment (FBT), i.e., ~20 sessions over 12 months. Weight and menstrual status were tracked at each therapy session throughout treatment. The primary outcome measures were weight (percent of expected for sex, age and height), and ROM.

**Results:**

Mean percent EBW at baseline was 82.0 (*SD* = 6.5). ROM was reported by 67.9% of participants (57/84), on average at 94.9 (*SD* = 9.3) percent EBW, and after having completed an average of 13.5 (*SD* = 10.7) FBT sessions (~70% of standard FBT). Compared to participants without ROM by treatment completion, those with ROM had significantly higher baseline Eating Disorder Examination Global scores (*p* = .004) as well as Shape Concern (*p* < .008) and Restraint (*p* < .002) subscale scores. No other differences were found.

**Conclusions:**

Results suggest that ROM occur at weights close to the reference norms for percent EBW, and that high pre-treatment eating disorder psychopathology is associated with ROM. Future research will be important to better understand these differences and their implications for the treatment of adolescents with AN.

## Background

Anorexia nervosa (AN) is a serious disorder that is associated with numerous medical and psychiatric complications [1-6]. AN typically onsets during adolescence [7], and is associated with delayed menarche in premenarchal females, or amenorrhea in postmenarchal females [8]. Amenorrhea, defined as the lack of three consecutive menstrual periods in postmenarchal females, is one of four criteria for AN in the *Diagnostic and Statistical Manual of Mental Disorders, Fourth Edition Text revision (DSM-IV-TR)*[9] and is currently considered a key clinical feature of AN. However, in anticipation of DSM-5, there has been considerable debate regarding whether amenorrhea is a useful criterion [10], especially as it applies to children and younger adolescents, females on contraceptives, and males. The *DSM-IV-TR* does not consider primary amenorrhea (i.e., failure of menses to start by age 15 while secondary sex characteristics such as breast development and growth of body hair are present, or within three years of the onset of secondary sexual characteristics) as part of the criteria for females with AN [11]. Further, a significant proportion of female adolescents with AN continue to menstruate at low weights [12], while minimal differences have been found between amenorrheic and menstruating individuals on clinically relevant variables such as length of illness (LOI), eating-related psychopathology, number of previous hospitalizations, and discharge weight [10].

Despite debate about its diagnostic utility, menstrual status as a measure of physiological health highlights the need for the early identification of serious medical issues [13], such as aberrations in metabolism, structural and functional brain alterations [14], and cardiovascular complications [15]. Importantly, individuals with amenorrhea achieve lower peak bone mass levels than age-matched controls [16,17]. It has been shown that pre-pubertal weight can be a useful tool to determine the weight at which resumption of menses (ROM) is likely to occur in adolescents with AN, highlighting the need to return to a weight trajectory preceding weight loss [18]. Conversely, ROM may be used as a key marker of recovery from AN as it is associated with return of physical health. For example, ROM in conjunction with weight gain has been found to correlate with increased bone mineral density in patients with AN [19,20]. It is important to consider several factors when estimating treatment goal weights in pediatric eating disorders. Goal weights typically take into account patient’s height, age, premorbid growth curve percentiles for height and weight, prior growth trajectory, growth potential, pubertal stage, anthropometric measurements and other physiological factors [1]. However, ROM does not only depend on reaching goal weight, as endocrine markers such as luteinizing hormone, estradiol, and triiodothyronine can also be useful in determining the onset or return of menses [3,18,21,22]. Notwithstanding, ROM, in conjunction with expected body weight (EBW), can serve as a yardstick to estimate goal weights for adolescent AN. In an elegant study of 100 adolescents with AN, menstruation resumed on average at 91.6% median weight for age and height. At one-year follow-up, 68% of participants resumed menses, and overall, 86% resumed menses within six months of achieving this weight [23]. It is of interest to note that 73% (16/22) of patients who remained amenorrheic did achieve a weight high enough to presumably resume menses. Using ROM in the context of EBW may predict more accurately return to health for patients with AN rather than using goal weight ranges alone. However, what differentiates patients at a minimally healthy weight with and without ROM remains unclear.

Overall, this study aimed to identify correlates of ROM that may assist clinicians with treatment planning. Our primary goal was to detect mean percent EBW and time point during a course of outpatient family-based treatment (FBT; the most well-established treatment for adolescent AN) [24] at which ROM occurs for adolescent AN. Our secondary goal was to examine demographic and psychosocial characteristics associated with ROM. As the relationship between amenorrhea and psychosocial variables remains unclear, we did not expect any differences between patients with and without ROM in terms of psychosocial characteristics at baseline evaluation [10,25]. We did, however, anticipate that patients with binge/purge behaviors would be more likely to resume menses during treatment than patients without such behaviors. Our hypothesis was informed by a study of 197 individuals treated at and inpatient eating disorders program comparing menstrual status (menstruating vs. amenorrheic) and AN subtype (restricting vs. binge/purge), which found a significantly higher number of patients who were menstruating to have the binge/purge subtype [10].

## Methods

### Participants

Potential participants were 225 females with AN, aged 11–20 years, presenting for treatment at the Eating Disorders Program at The University of Chicago between September 2001 and September 2011. Of those, 47 were prepubertal or presented with primary amenorrhea, 56 had regular or irregular periods, 27 were taking oral contraceptives, and 11 did not provide this information. Therefore, 84 females with secondary amenorrhea, meeting *DSM-IV* criteria for AN or partial AN were included in the current study. Partial AN was defined as <90% EBW and was included as many young patients with eating disorders do not fit criteria used for adults, while being at risk for developing significant health complications following less severe weight loss than might be typical among adult populations [26-28]. EBW is one method used to set appropriate treatment goal weights in eating disorder patients [8], and defines the optimal weight for height and/or age in order to achieve healthy nutritional status with the lowest rate of mortality [29]. Calculation of percent EBW is not standardized across studies with various methods leading to sizable discrepancies within cases [30]. For the present study the Body Mass Index (BMI) Method was used, which for a given subject is based on height, age, and sex. The 50th percentile BMI for exact age and height at presentation, as measured by the Centers for Disease Control and Prevention growth charts [31], was used for this calculation (%EBW = BMI/50th percentile BMI for age and height x 100). Growth over the course of treatment was measured to obtain accurate EBW at ROM and end of treatment time points [32].

### Measures

The Eating Disorder Examination (EDE) is a semi-structured diagnostic interview assessing the frequency and severity of eating disorder behaviors and cognitions [33]. The EDE generates four subscale scores as well as a composite global score. Scores range from 0 to 6, with higher scores indicating greater severity of eating disorder symptoms. The EDE was used in this study to generate diagnoses, determine baseline menstrual status, and assess eating disorder symptoms. Demographic information and history of prior medical and/or psychiatric hospitalizations were obtained from a diagnostic interview. Weight and height were measured on a calibrated scale and stadiometer, respectively. In this study ROM was defined as at least one spontaneous menstrual cycle while in treatment.

### Procedure

The Institutional Review Board at The University of Chicago approved this research. Written assent and/or consent were obtained from each patient and parent/guardian, where appropriate (i.e., assent if patient was <18 years). All patients received manualized FBT administered by a trained clinician in our program. FBT has received robust research support [34], and is a manualized treatment that focuses on enlisting the parents, guided by the therapist, in facilitating weight restoration for the ill child, and is typically delivered in 20 sessions over 12 months [24]. Patients, in light indoor clothing, were weighed by their therapist at the outset of each treatment session and asked to self-report menstrual status.

### Statistical analysis

Descriptive statistics were used to examine percent EBW and FBT session at which ROM occurred. Independent samples t-tests were used to compare patients with full versus partial AN in age, illness duration, eating disorder psychopathology, and weight gained during treatment. AN cases were compared to partial AN to test whether (a) these two groups were broadly comparable, and (b) ensure that differences between patients with or without ROM were not due to diagnosis. Chi-square tests were used to examine differences between AN and partial AN in terms of previous medical and/or psychiatric hospitalizations and ethnicity. In addition, independent samples t-tests were conducted to determine baseline differences in age, percent EBW, BMI, LOI, average amount of weight gained during treatment, and eating disorder pathology between patients with or without ROM. Chi-square tests were used to compare patients with or without ROM in terms of previous medical and/or psychiatric hospitalizations and ethnicity, and to determine whether patients with bingeing and/or purging behaviors at baseline were any more or less likely to resume menses during treatment than patients with a restricting subtype.

In an exploratory analysis, patients who achieved ROM were compared across a range of physiological and psychosocial variables to a subset of patients who gained the appropriate amount of weight to expect menses to return but failed to resume menses.

## Results

### Participant characteristics

Mean age of participants was 15.1 (*SD* = 2.2) years. Seventy-seven percent self-identified as Caucasian, 12% as Hispanic, 2% as African American, 4% as Asian, and 5% as ‘other’. Fifty-three (63%) patients met full *DSM-IV-TR* criteria for AN, while 31 (37%) were categorized as partial AN. There were no statistically significant differences between patients with AN and partial AN in age, ethnicity, LOI, and EDE subscale and global scores (all *p*’s > .13). As expected, baseline percent EBW was lower for patients with AN than for those with partial AN (*M* = 80.3, *SD* = 7.1 vs. *M* = 84.9, *SD* = 4.0, *t*(82) = −3.31, *p* < .001), while patients with AN gained more weight (in lbs.) from baseline to ROM compared to partial AN, even after controlling for baseline percentage EBW (*M* = 15.5, *SD* = 10.3 vs. 10.6, *SD* = 9.4, *F*(2) = 4.74, *p* = .01). There was no difference in terms of previous hospitalization for patients with AN compared to those with partial AN (χ^2^(1) = 4.05, *p* = .06).

### Resumption of menses

Mean percent EBW at baseline was 82.0 (*SD* = 6.5), with 67.9% (57/84) having ROM. Average percent EBW at which ROM occurred was 94.9% (*SD* = 9.3) and average weight gain required for ROM during treatment was 14.7 lbs. (*SD* = 10.8). (A table with the exact percent EBW at which each participant experienced ROM is available from the corresponding author upon request). Mean session number at which ROM occurred was 13.5 (*SD* = 10.7), i.e., ~70% of standard FBT. There were no differences in baseline age, ethnicity, or LOI between patients with and without ROM. ROM was not associated with percent EBW at baseline (*t*(82) = −0.77, *NS*), meaning that patients who weighed less at baseline were no less likely to resume menses during treatment than patients at higher initial weights. Patients with partial AN were more likely than full AN to resume menses during treatment (χ^2^(1) = 3.82, *p* = .04). Patients with binge/purge behaviors were no more likely to resume menses than patients without those behaviors at initial presentation (χ^2^(1) = 0.90, *NS*). Patient characteristics for those with and those without ROM are summarized in Table 1.

**Table 1:** **Baseline and End-of-Treatment Participant Characteristics with/without ROM ( *M, SD *)**

**Variable**	**ROM (n = 57)**	**No ROM (n = 27)**	***t***	***p***
Age in Years	15.21 (2.18)	14.99 (2.29)	-0.44	.66
Percent EBW	82.39 (6.67)	81.2 (6.29)	-0.77	.44
BMI	16.50 (1.29)	16.32 (1.44)	-0.58	.57
Duration ill in months	16.15 (17.23)	12.21 (13.05)	-0.92	.36
EDE Weight Concern	2.35 (1.68)	1.75 (1.51)	-1.45	.15
EDE Shape Concern	2.89 (1.78)	1.67 (1.73)	-2.75	**.008**
EDE Restraint	2.94 (1.59)	1.59 (1.86)	-3.20	**.002**
EDE Eating Concern	1.89 (1.47)	1.40 (1.45)	-1.33	.19
EDE Global Score	2.47 (1.44)	1.40 (1.41)	-2.97	**.004**
**Ethnicity**	**ROM (Percent)**	**No ROM (Percent)**	***χ*** ***²***	***p***
Caucasian	80.7	70.4	1.12	.22
Hispanic	10.5	14.8	0.32	.41
African-American	0.0	7.4	4.31	.10
Asian	1.8	7.4	1.70	.24
Other/Not Specified	7.0	0.0	1.99	.21
Previous hospitalizations	28.3	24.0	0.16	.79
Restricting vs. Binge/Purge	60.0/40.0	70.4/29.6	0.90	.34

Key: ROM = Return of Menses; EBW = Expected Body Weight; BMI = Body Mass Index; EDE = Eating Disorder Examination; EOT = End-of-Treatment.

### ROM Group versus no-ROM Group

Patients with ROM gained more weight during treatment than those without ROM, but this difference was not statistically significant (Mean = 94.9% EBW, *SD* = 9.3 vs. 91.6% EBW, *SD* = 8.9). Patients with ROM had significantly higher EDE Global Scores (*p* < .004), as well as Shape Concern (*p* < .008) and Restraint (*p* < .002) subscale scores compared to those without ROM. No other differences were found between these two groups.

### ROM versus no-ROM in patients with ≥ 95% EBW

Of the 27 patients who did not resume menses, 10 achieved ≥95% of EBW. In an exploratory analysis we compared this subset of patients (*n* = 10) to those with ROM after reaching 95% of EBW on LOI, percent of EBW at baseline, amount of weight gained during treatment, and EDE subscale and global scores. Mann–Whitney *U* tests revealed significantly higher EDE Shape Concern, Restraint, and Global Scores for the group with ROM. There were no other differences between these two groups (See Table 2).

**Table 2:** Baseline participant characteristics with ROM versus participants who reached 95% of EBW without ROM (M, SD)

**Variable**	**ROM (n = 57)**	**95% no ROM (n = 10)**	***z***	***p***
Age in years	15.21 (2.18)	14.15 (2.04)	-1.17	.24
Percent EBW	82.39 (6.67)	85.51 (4.19)	1.53	.13
Weight gained during Tx (lbs.)	14.67 (10.83)	16.14 (6.57)	1.06	.29
Duration ill in months	16.15 (17.23)	12.67 (7.55)	0.23	.82
EDE Weight Concern	2.35 (1.68)	1.37 (0.81)	-1.47	.14
EDE Shape Concern	2.89 (1.78)	0.81 (0.88)	-3.18	**<.01**
EDE Restraint	2.94 (1.59)	0.73 (1.30)	-3.17	**<.01**
EDE Eating Concern	1.89 (1.47)	1.16 (1.39)	-1.34	.18
EDE Global Score	2.47 (1.44)	1.01 (0.77)	-2.77	**<.01**
**Ethnicity**	**Percent**	**Percent**	***χ*** ***²***	***p***
Caucasian	80.7	70.0	0.59	.34
Hispanic	10.5	10.0	0.03	.72
African-American	0.0	10.0	5.79	.15
Asian	1.8	10.0	2.00	.28
Other/Not Specified	7.0	0.0	0.75	.52
Previous Hosp (Med/Psych)	28.3	33.3	0.10	.52

Key: ROM = Return of Menses; EBW = Expected Body Weight; EDE = Eating Disorder Examination.

## Discussion

This study examined ROM in adolescent AN in relation to percent EBW to estimate appropriate goal weights for this patient population. All subjects were undergoing outpatient FBT. We also examined demographic, physiological, and psychosocial characteristics associated with ROM. ROM occurred, on average, at 95% EBW suggesting that as a minimum this weight target is necessary to restore menses in adolescents, at least for those receiving an outpatient course of FBT. Our findings support those of a prior study which used pelvic ultrasound to demonstrate that ovarian and uterine maturity in adolescent AN occur at 96.5% EBW [35]. Together, these findings may suggest that previous goal weights of 90% EBW for this population might be too low to support ROM [23]. In a recent study [1], it was suggested that BMI percentiles between 14 and 39 could be used to estimate treatment goal weight. Others reported that about one third of AN adolescents with ROM did so at weights above population averages [18], and in a comparison of low weight AN patients without and with menses, the latter consumed a more nutritionally ‘balanced’ diet than their amenorrheic counterparts [36,37]. In our own exploratory analyses, about one third of patients without ROM achieved 95% EBW or higher. Individual differences in weight trajectories should be taken into account, as these data seem to indicate, as many patients may have higher individual “setpoint” weights than the average EBW calculations provide. These discrepancies, however, may also be related to weight maintenance necessary to restore menstrual function in adolescent AN as some patients will only resume menses at weights >2 kg’s above the level that supported menses prior to the onset of amenorrhea [23]. Together, these findings speak to why there is such a wide range of weights at which menses resume.

It is of interest, though not surprising, that our results differ from the often used adult standard of 90% EBW as a treatment goal for AN [38], highlighting the need to determine goal weights that are specific to adolescents. For example, regular assessment, review, and adjustments of goals weights are needed for this population, as these are “moving targets” when growth in both height and weight are expected during the pubertal years. Contrary to our hypothesis, those with ROM reported *more* severe baseline EDE psychopathology than those without ROM. This is interesting as these groups were not shown to have other differences in baseline characteristics, including percent EBW and duration of illness. It is possible that FBT for adolescent AN with more severe eating disorder symptomatology differs from those who endorse fewer symptoms [39]. Although quite speculative, parents of adolescents who are more impaired by their eating disorder are perhaps more likely to appreciate the urgency and severity of their child’s illness, and perhaps more engaged in FBT. It is reasonable to assume that when the disorder appears ‘less severe’ (i.e., physical and psychological consequences are not as obvious and do not appear imminently threatening), families may fail to fully commit to FBT. There may also be a distinction in severity of the physical versus psychological symptoms of AN that could account for differences between those with or without ROM.

Some strengths and limitations should be noted. This study is among the first to examine the weight (% EBW) at which ROM occurs after a course of a manual-based treatment modality (FBT). However, there was great variability in the number of treatment sessions at which menses returned, demonstrating that some patients recommenced menses after only a few sessions, while others required much more treatment. Another strength of this study is the examination of baseline psychological variables that correlate with ROM during FBT. Limitations include a modest sample size comprised mostly of Caucasian patients, thereby restricting the generalizability of our findings. In addition, there are limitations to using EBW based on the CDC reference norms in children and adolescents, which rely on cross-sectional data and do not take into account the timing of puberty [30]. Girls who reach puberty earlier or later than average may not follow the same pattern in height and weight percentiles. In addition, menstrual status data were collected using semi-structured interviews, questionnaires, or self-report. Adolescents with AN may choose not to fully disclose their current menstrual status, or may not remember their menstrual history accurately. In addition, irregular menstrual cycles are common among adolescent females, especially during the first two years post-menarche [40]. As such this may further complicate the reason for the amenorrhea. No physiological or parent-as-informant measures against which to validate self-reported menstrual status were systematically employed. Future studies could use more objective measures to corroborate ROM, e.g., luteinizing hormones, follicle-stimulating hormones, estradiol hormonal levels, and pelvic ultrasound [1,35]. While all patients in this study were treated with FBT, it does not inform us whether these findings would generalize to patients receiving other forms of treatment. In addition, information on menstrual threshold weight was not available. It is possible that patients who lost their menses at higher EBW percentages will need to gain more weight to achieve ROM [1].

## Conclusions

Findings from this study underscore the need for full weight restoration based on EBW as a primary treatment goal for the pediatric eating disorder populations. Several factors are important to consider in setting appropriate goal weights during weight restoration, e.g., past height and weight percentiles, premorbid weight using menstrual threshold or weight trajectory over time (if applicable), sexual maturity, and age. When information concerning the patient’s weight history is unavailable or conflicting, our results suggest that aiming for at least 95% EBW is an appropriate target for these patients in outpatient treatment. Menstrual status is a frequently used measure of severity of illness in eating disorders [41], making ROM an objective way to join percent EBW with a physical indicator of biological health. Utilizing percent EBW in this way can potentially lead to more accurate predictions of goal weights during treatment. This does not negate that percent EBW at which ROM occur differs among patients, with some menstruating at body weights lower than 95% EBW and others at body weights higher than this threshold. Therefore, other clinical practice parameters, such as pelvic maturity with ultrasonography and hormonal status, might be more useful than percent EBW to identify target weights for the ROM. Moreover, with growth and development and over time, the EBW will change.

This is the first study to examine when ROM occurs during FBT, and although tentative, it does provide clinicians, and patients and families treated with FBT, a clinical benchmark in that ROM occurred at 70% of the standard number of sessions in this treatment. FBT is manualized, has robust research support [24,34], and eliminates the possibility of treatment group effects in this sample. Consequently, we were able to identify baseline psychosocial differences between patients with and without ROM during a course of FBT. For now, this finding is speculative, and potential reasons for this association might be the large number of adolescent patients who fail to report overvaluation of shape and weight and instead endorse overvaluation of eating control. It should also be noted that it is possible that feeling fat, or perceiving/experiencing bloating in the pre-menstruation period, or experiencing these feelings with weight gain alone, my intensify shape concern. Future research will be vital to better understand these differences and their implications for the treatment of adolescents with AN.

## Competing interests

This study was supported by grants MH070620 and MH093768 (Dr Le Grange) from the National Institutes of Health. Dr. Le Grange receives royalties from Guilford Press and Drs. Le Grange and Loeb receive honoraria from the Training Institute for Child and Adolescent Eating Disorders, LLC.

## Authors’ contributions

JF, KA, and DLG conceived of the study, and participated in its design, coordination, and helped draft the manuscript. JF performed the statistical analysis, while AG, KL, MB and DK participated in drafting the manuscript. All authors read and approved the final manuscript.
